# Histology of neovascular myopic macular degeneration

**DOI:** 10.1038/s41598-021-01500-2

**Published:** 2021-11-09

**Authors:** Shefali B. Jonas, Songhomitra Panda-Jonas, Jost B. Jonas, Rahul A. Jonas

**Affiliations:** 1 Medizinische Hochschule Hannover, Hannover, Germany; 2 Privatpraxis Prof Jonas und Dr Panda-Jonas, Heidelberg, Germany; 3 Department of Ophthalmology, Medical Faculty Mannheim of the Ruprecht-Karls-University of Heidelberg, Mannheim, Germany; 4 Institute of Molecular and Clinical Ophthalmology Basel, Basel, Switzerland; 5 Department of Ophthalmology, Medical Faculty, University Hospital Cologne, University of Cologne, Cologne, Germany

**Keywords:** Retinal diseases, Macular degeneration

## Abstract

To assess the histological correlate of neovascular or exudative myopic macular degeneration (nMMD) in highly myopic human eyes, we examined histomorphometrically histologic sections of enucleated eyes of Caucasian patients. The study included 284 eyes (age: 61.9 ± 13.7 years; range: 24–89 years; axial length: 25.5 ± 3.1 mm; range: 20–37 mm). An nMMD was detected in 5 eyes (axial length: 29.6 ± 2.6 mm; range: 26.0–31.0 mm). All these eyes showed within or close to the nMMD a macular Bruch’s membrane (BM) defect, fibrous tissue with erythrocyte-filled blood vessels, and proliferations of irregularly pigmented and irregularly piled-up retinal pigment epithelium (RPE) cells each of which was connected with a curled-up, PAS (Periodic-Acid-Shiff)-positive membrane. The nMMD lesions were covered by proliferated RPE cells. RPE cells were not detected within the retina. In binary regression analysis, a higher nMMD prevalence was associated with a higher prevalence of macular BM defects (odds ratio: > 1000; *P* < 0.001), while the association with axial length was not significant (*P* = 0.43) in that model. After adjustment for the presence of macular BM defects, the nMMD prevalence was not associated with BM thickness (measured at the posterior pole, equator-posterior pole midpoint, equator and shortly posterior to the ora serrata) (*P* = 0.10; *P* = 0.87; *P* = 0.40; and *P* = 0.36, respectively), RPE cell layer thickness (*P* = 0.83; *P* = 0.79; *P* = 0.31; *P* = 0.38, resp.), RPE cell density (*P* = 0.56; *P* = 0.91; *P* = 0.47; *P* = 0.87, resp.), choriocapillaris thickness (*P* = 0.47; *P* = 0.93; *P* = 0.41; *P* = 0.75, resp.), and choriocapillaris density (*P* = 0.99; *P* = 0.94; *P* = 0.17; *P* = 0.97, resp.). The results suggest that nMMD is characterized by a fibrous pseudo-metaplasia of the RPE and is strongly associated with macular BM defects, without other detected histomorphometric differences in thickness or density of the RPE, BM, and choriocapillaris.

## Introduction

Exudative or neovascular myopic macular degeneration (nMMD), also called myopic choroidal neovascularization, is part of a panoply of features characterizing myopic macular degeneration. Treatment consists of the intravitreal application of vascular endothelial growth factor (VEGF) antibodies, such as ranibizumab, aflibercept and bevacizumab^1–3^. Clinical risk factors for nMMD include long axial length within the range of high axial myopia, female sex, older age, and potentially a further axial elongation^4–7^. It has remained unclear whether other features of nMMD additionally play a role. These features include choroidal thinning, enlargement of Bruch’s membrane (BM) opening of the optic nerve head, enlargement of the optic disc, development and enlargement of parapapillary gamma zone and delta zone, increase in the disc-fovea length with a straightening of the papillo-macular retinal vessels and retinal ganglion cell axons, development and enlargement of secondary BM defects in the macular region, development of a macular ridge or a dome-shaped macula^4,5,8–11^. Furthermore, the histologic correlate of nMMD has only scarcely been described so far^12^. We therefore conducted this study to assess and describe histo-pathological findings of nMMD in enucleated, highly axially elongated, human globes and to search for associated histological factors. The findings may be of help to further characterize nMMD, to describe associated, and potentially predisposing, factors, and to discuss the etiology of nMMD and the process of axial elongation in general.

## Methods

The histomorphometric investigation included enucleated human globes which had been removed due to diagnoses such as malignant choroidal melanomas and painful end-stage glaucomas. The study was approved by the Medical Ethics Committee II of the Medical Faculty Mannheim of the Heidelberg University. It confirmed that the study adhered to the Declaration of Helsinki and that all methods were performed in accordance with the relevant guidelines and regulations. The necessity of an informed consent by the patients had been waived by the Medical Ethics Committee, since the globes had been enucleated up to 50 years before start of the study. An informed consent was thus not obtained from the study participants. Some of the eyes have been included in previous studies addressing different questions^13,14^. Eyes with a choroidal tumor at the posterior pole were excluded from the study.

As described previously, the eyes were fixed in a solution of 4% formaldehyde and 1% glutaraldehyde shortly after enucleation. After measuring the sagittal, vertical and horizontal globe diameters, we removed an 8 mm thick middle segment running through the optic nerve head and the pupil, dehydrated the segment in alcohol and imbedded it in paraffin. In the group of eyes with malignant choroidal melanomas, the location of the tumor determined the meridian of the segment, while in all other eyes, the orientation of the segment was horizontal. Out of the segments, we prepared histologic sections with a thickness of 8 µm. The sections were stained by the Periodic-Acid-Shiff (PAS) method or with hematoxylin eosin. For all eyes, we took one histological section, running through the central part of the pupil and optic nerve head, for further assessment.

Upon light microscopy, all globes were examined for the presence of nMMD, defined by a tissue layer between the photoreceptor layer and BM. Using an in-built micrometer scale in the objective of the microscope, we measured histomorphometrically the thickness of BM and of the choriocapillaris and we determined the density of the retinal pigment epithelium (RPE) cells and choriocapillaris vessels at the posterior pole, at the midpoint between the equator and the posterior pole, at the equator, and at a location just posterior to the ora serrata. In addition, we searched for secondary defects of BM.

Using a statistical software program (SPSS for Windows, version 25.0; IBM-SPSS, Chicago, Illinois, USA), we calculated the mean values and standard deviations of the main outcome parameters. Using Student’s t-test or the Mann–Whitney test for unpaired samples, we determined the significance of differences in these parameters between the study group consisting of eyes with nMMD and a control group of eyes without nMMD. We assessed associations between the nMMD prevalence and other parameters in a binary regression analysis, first in a univariate mode, then in a multivariable manner. The list of independent parameters consisted of those parameters, which were significantly correlated with the nMMD prevalence in the univariate analysis. We calculated the odds ratios (ORs) and their 95% confidence intervals (CIs). A *P* value of < 0.05 (two-sided) was considered to be statistically significant.

## Results

The study included 284 eyes of 284 patients with an age of 61.9 ± 13.7 years (median: 63 years; range: 24–89 years). Mean axial length was 25.5 ± 3.1 mm (median: 24.0 mm; range: 20–37 mm). An nMMD was detected in 5 eyes with a mean axial length of 29.6 ± 2.6 mm (range: 26.0–31.0 mm). Histomorphometric measurements of the thickness and density of the choriocapillaris, BM and RPE were available for 79 eyes (61.0 ± 17.1 years).

**Figure 1:**
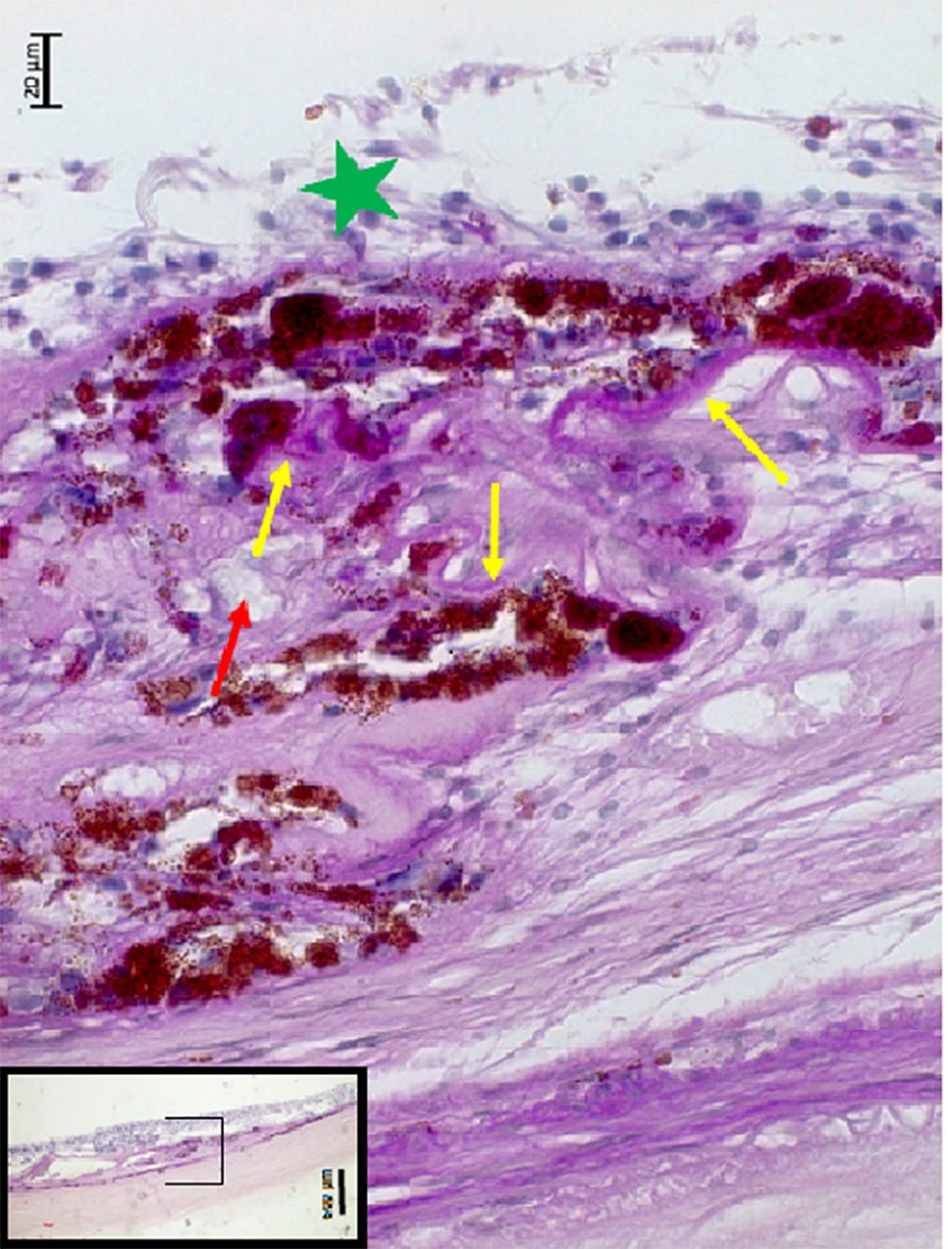
Histophotograph showing a neovascular myopic macular degeneration with irregular proliferations of irregularly pigmented retinal pigment epithelium cells, each of which connected with a PAS (Periodic-Acid-Shiff)-positive, crumbled basal membrane (yellow arrows), retinal capillaries (red arrow), and fibrous tissue, located beneath a disarranged retina (green asterisks).

**Figure 2:**
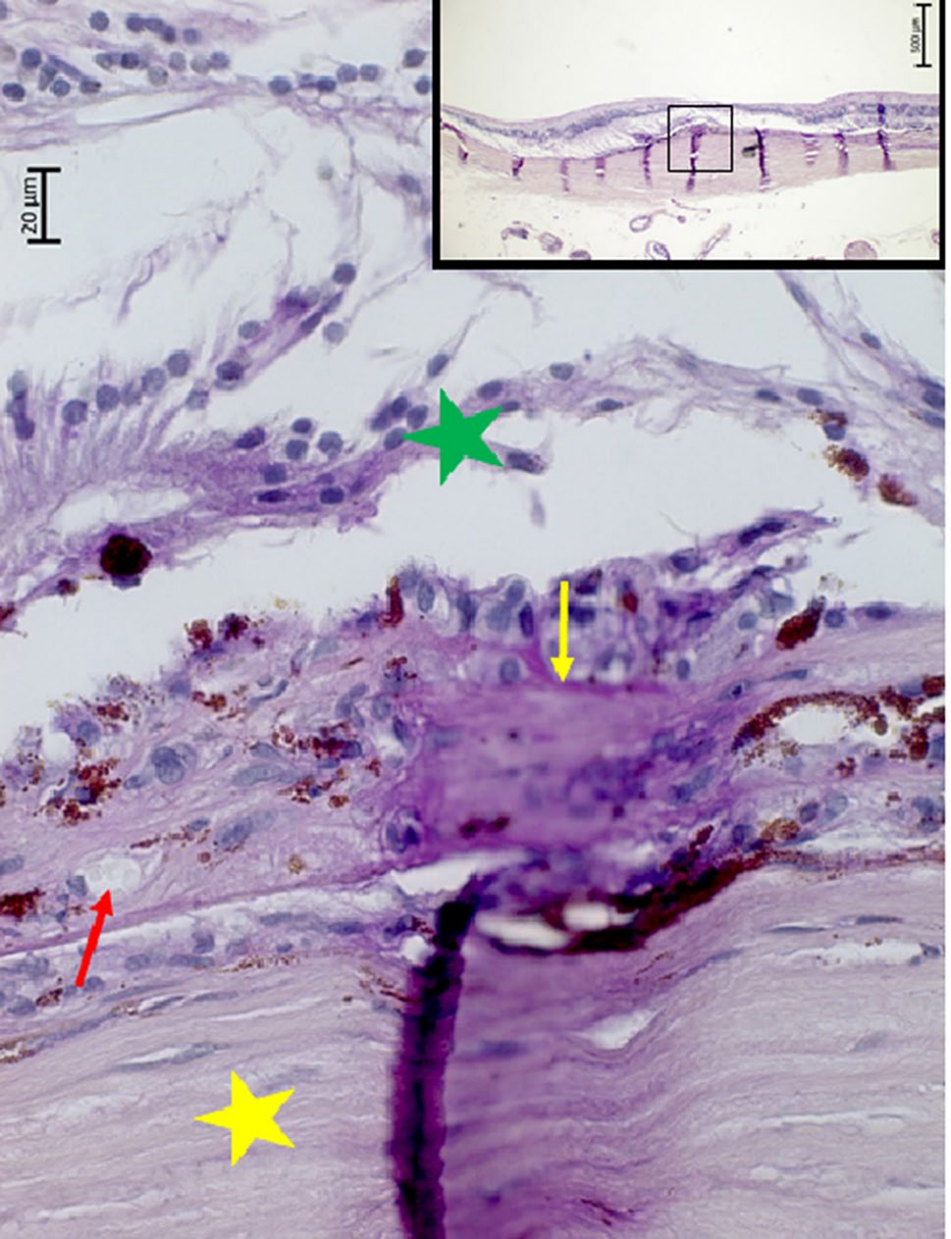
Histophotograph showing a neovascular myopic macular degeneration with irregular proliferations of irregularly pigmented retinal pigment epithelium cells, connected with a PAS (Periodic-Acid-Shiff)-positive, crumbled basal membrane (yellow arrow), retinal capillaries (red arrow), and fibrous tissue, located beneath a disarranged retina (green asterisks); yellow asterisks: sclera.

**Figure 3:**
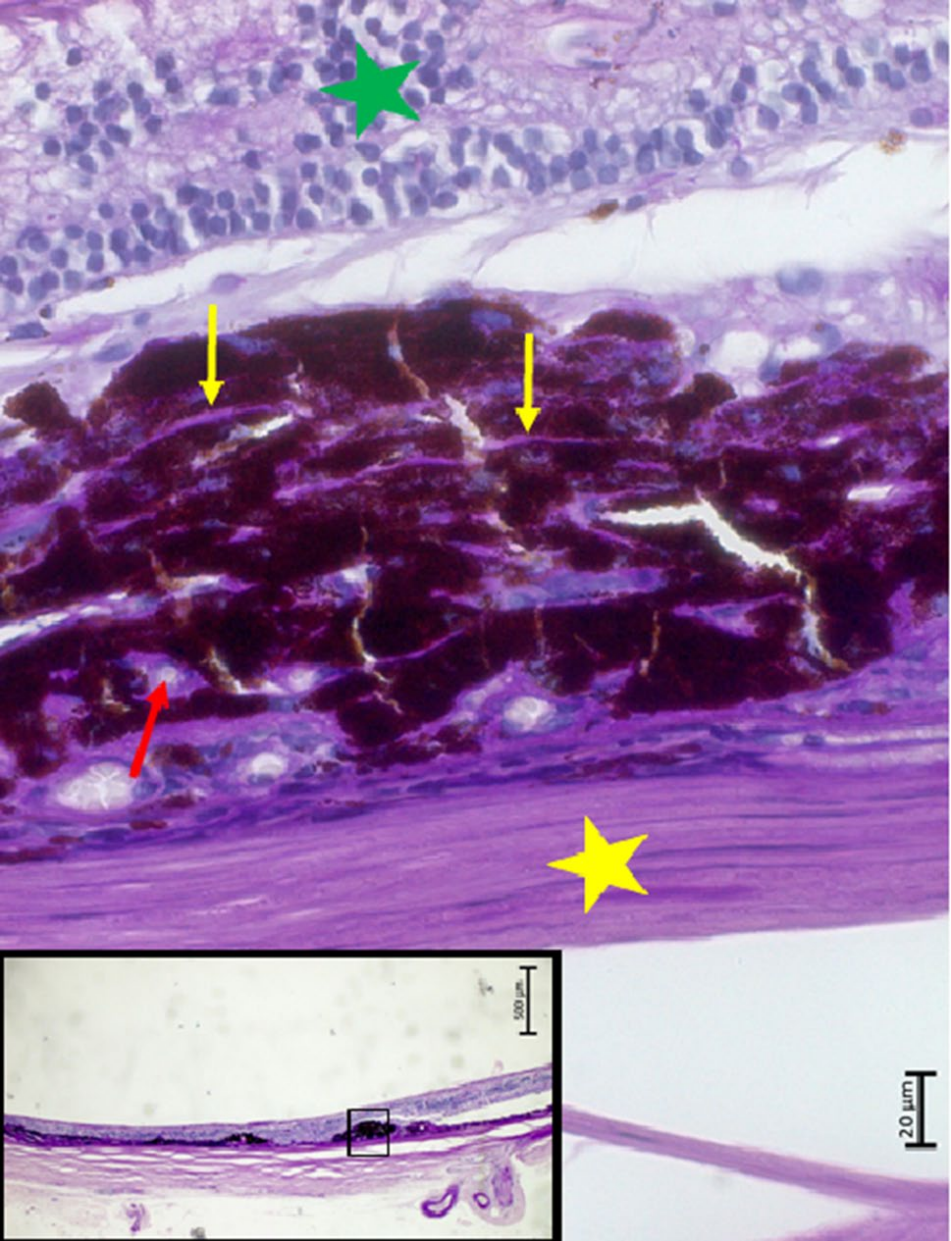
Histophotograph showing a neovascular myopic macular degeneration with irregular proliferations of irregularly pigmented retinal pigment epithelium cells, connected with a PAS (Periodic-Acid-Shiff)-positive, crumbled basal membrane (yellow arrows), retinal capillaries (red arrow), and fibrous tissue, located beneath a disarranged retina (green asterisks); yellow asterisks: sclera.

**Figure 4:**
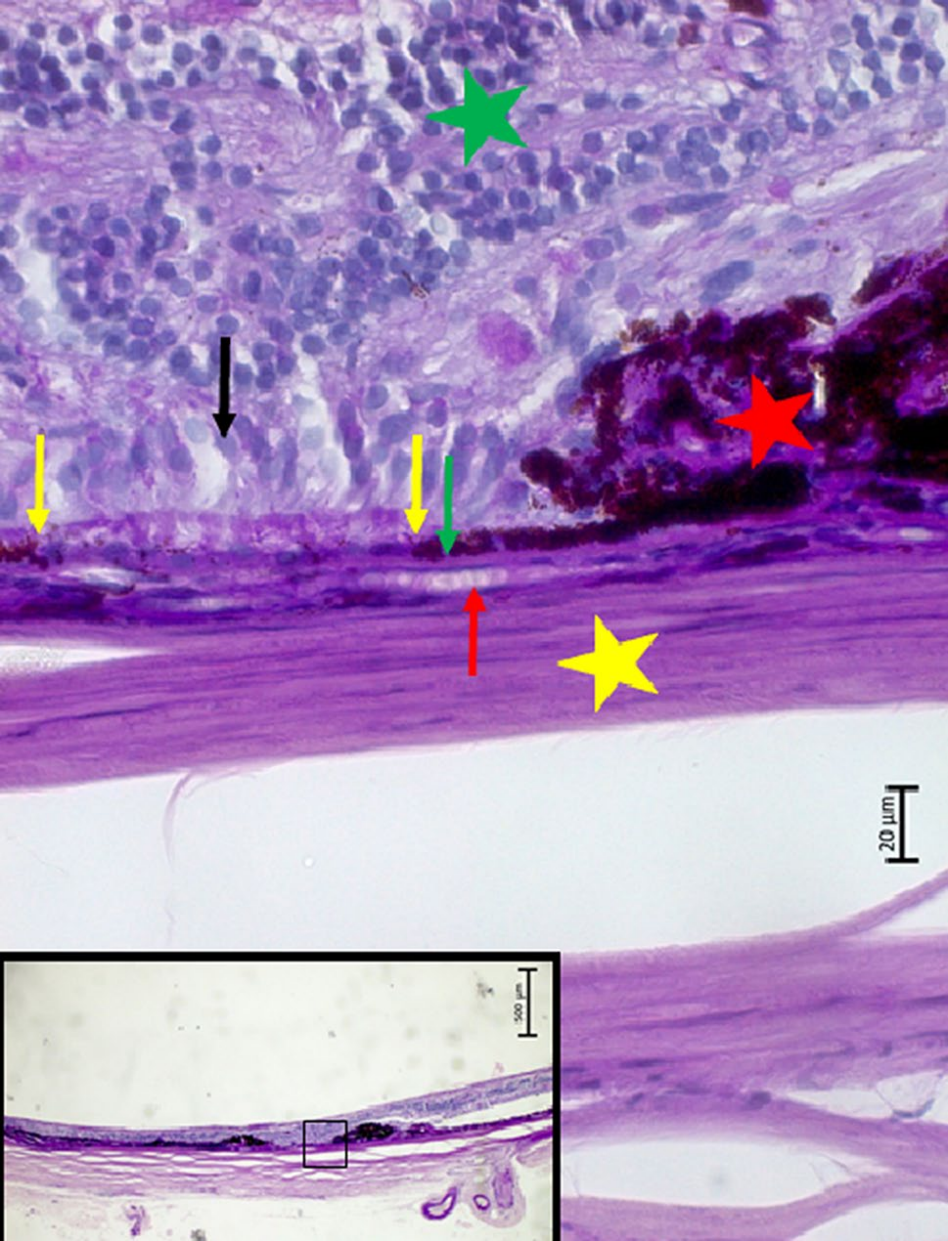
Histophotograph showing an eye with neovascular myopic macular degeneration with irregular proliferations of irregularly pigmented retinal pigment epithelium cells (red asterisks); between yellow arrows: depigmented retinal pigment epithelium, resting on intact Bruch’s membrane (green arrow) and choriocapillaris (red arrow); black arrow: photoreceptors; green asterisks; retina; yellow asterisks: sclera.

All five eyes with nMMD showed proliferations of irregularly pigmented and irregularly piled-up RPE cells each of which was connected with a PAS-positive, curled-up membrane (Figs. 1, 2, 3, 4). This fibrous tissue contained erythrocyte filled blood vessels in the form of capillaries or small venule-like blood vessels. In all five eyes, the nMMD was close to, or included, a macular BM defect. The photoreceptor layer, if present at all, did not show photoreceptor outer segments. In none of the five eyes, proliferated RPE cells were detected within the retina. The choroid as a whole and the choriocapillaris within the area of the nMMD did not show abnormalities as compared to eyes with similar axial length and not having an nMMD. In the vicinity of the nMMDs, the RPE layer contained regularly pigmented and arranged RPE cells connected with an intact BM of normal thickness. The choriocapillaris was continuous and showed a normal thickness and density of blood vessels. In one eye, which contained two nMMDs, the RPE in the region between both nMMDs was segmentally depigmented. It was located on a normal appearing BM of a normal thickness with a normal appearing choriocapillaris (Fig. 4).

Comparing the study group of 5 eyes with nMMD with the group of the eyes without nMMD revealed, that the nMMD group had a significantly older age (*P* = 0.005), longer axial length (*P* = 0.005) and a higher prevalence of macular BM defects (*P* < 0.001) (Table 1). All five eyes with nMMDs had a collateral BM defect. In a binary regression analysis, the prevalence of nMMD was associated only with a higher prevalence of macular BM defects (OR: > 1000; *P* < 0.001), while the associations with axial length (*P* = 0.43) and age (*P* = 0.28) were not significant in that model.

**Table 1:** Histomorphometric measurements (mean ± standard deviation) of eyes with neovascular myopic macular degeneration (nMMD) versus eyes with nMMD.

Parameter	Myopic macular degeneration	No myopic macular degeneration	*P* value
n	5	279	–
Age (years)	81.5 ± 5.0	61.6 ± 13.6	0.02
Axial length (mm)	29.6 ± 2.6	25.4 ± 3.1	0.005
Bruch’s membrane defect presence	5 (100%)	14 (5.1%)	< 0.001
Bruch’s membrane thickness (µm), posterior pole	2.2 ± 0.7	2.6 ± 0.9	0.20
Bruch’s membrane thickness (µm), midpoint posterior pole to equator	2.6 ± 0.2	2.5 ± 1.0	0.99
Bruch’s membrane thickness (µm), equator	0.8 ± 0.4	0.8 ± 0.3	0.83
Bruch’s membrane thickness (µm), shortly posterior to the ora serrata	3.3 ± 1.1	2.5 ± 1.2	0.17
Retinal pigment epithelium (RPE) layer thickness (µm), posterior pole	8.1 ± 2.4	6.8 ± 2.5	0.84
RPE layer thickness (µm), midpoint posterior pole to equator	5.7 ± 1.4	5.5 ± 2.4	0.90
RPE layer thickness (µm), equator	5.2 ± 1.6	5.3 ± 2.1	0.24
RPE layer thickness (µm), shortly posterior to the ora serrata	6.1 ± 1.5	5.6 ± 2.5	0.51
RPE cell density, posterior pole	38 ± 8	30 ± 7	0.17
RPE cell density, midpoint between posterior pole to equator	20 ± 6	24 ± 9	0.34
RPE cell density, equator	11 ± 2	20 ± 9	0.045
RPE cell density, shortly posterior to ora serrata	25 ± 5	25 ± 7	0.77
Choriocapillaris thickness (µm), posterior pole	3.8 ± 1.0	4.6 ± 2.4	0.30
Choriocapillaris thickness (µm), midpoint posterior pole to equator	5.9 ± 1.3	3.8 ± 2.4	0.79
Choriocapillaris thickness (µm), equator	3.7 ± 2.2	2.8 ± 1.9	0.52
Choriocapillaris thickness (µm), shortly posterior to the ora serrata	3.8 ± 3.9	3.9 ± 2.7	0.64
Choriocapillaris density (µm/300 µm), posterior pole	205 ± 107	187 ± 61	0.12
Choriocapillaris density (µm/300 µm), midpoint posterior pole to equator	238 ± 32	168 ± 77	0.82
Choriocapillaris density µm/300 µm), equator	165 ± 45	144 ± 86	0.49
Choriocapillaris density µm/300 µm), shortly posterior to the ora serrata	140 ± 81	168 ± 83	0.59

After adjustment for the presence of macular BM defects, the nMMD prevalence was not significantly associated with the thickness of BM (as measured at the posterior pole, the equator-posterior pole midpoint, the equator and shortly posterior to the ora serrata) (*P* = 0.10; *P* = 0.87; *P* = 0.40; and *P* = 0.36, respectively), thickness of the RPE cell layer (*P* = 0.83; *P* = 0.79; *P* = 0.31; and *P* = 0.38, resp.), density of the RPE (*P* = 0.56; *P* = 0.91; *P* = 0.47; and *P* = 0.87, resp.), thickness of the choriocapillaris (*P* = 0.47; *P* = 0.93; *P* = 0.41; and *P* = 0.75, resp.), and density of the choriocapillaris (*P* = 0.99; *P* = 0.94; *P* = 0.17; and *P* = 0.97, resp.).

## Discussion

In this histomorphometric study on human enucleated globes, histological signs of nMMD were found in five eyes. These signs included a fibrous pseudo-metaplasia of the RPE, forming a fibrous tissue beneath the photoreceptor layer. It contained proliferated, irregularly pigmented RPE cells with each cell connected with a PAS-positive basal membrane. These PAS-positive membranes were crumbled and curled up, and the RPE cells were irregularly arranged in several layers. The subretinal fibrous tissue contained perfused blood vessels such as capillaries and few larger-scaled blood vessels. In all five eyes, the nMMD was close to a macular BM defect, and in multivariable analysis, the nMMD prevalence was associated with a higher prevalence of macular BM defects, while the association with longer axial length was not significant in that model. The photoreceptor layer, if present at all, did not show outer segments.

The observations made in our study agree with the histological description of a nMMD made by Grossniklaus in his pioneering study in 1992 who found “subretinal neovascularization with a fibrovascular scar, Fuchs’ spot, or lacquer cracks in 5.2% of the eyes” of his study population^12^. Grossniklaus additionally stated that “Fuchs’ spot represents subretinal or intraretinal migration of retinal pigment epithelium accompanying choroidal neovascularization with or without retinal vascular anastomosis.” A defect in BM was described to occur in association with lacquer cracks^12^. The new findings in our study are that the RPE proliferations occur, similar as the proliferations of the lens epithelium in the case of secondary cataract, in association with a basal membrane, so that it can be considered to be a fibrous pseudo-metaplasia of the RPE^15^. Of interest may also be that the nMMD lesions contained perfused blood vessels. Although it was not known when the nMMDs had developed in our study population, clinical experience suggests that the activity of nMMDs subsides after few weeks to months^1^. The presence of perfused blood capillaries in the presumably old nMMD lesions in our study population suggests an active role of the blood vessels in the lesion. Interestingly, definite signs of a tissue edema within the nMMD lesion were not found. It may be a contrast to macular lesions in exudative or neovascular age-related macular degeneration, in which an edema is typically found in OCT-based histology. One has to take into account however, that the histologic processing with the development of artificial tissue clefts may make the light microscopical detection of true tissue edema difficult.

Another finding of interest in our study was the concurrence of nMMDs with macular BM defects. In multivariable analysis, a higher nMMD prevalence was associated with a higher prevalence of BM defects, while the correlation with longer axial length had lost its statistical significance in that model. Since a BM defect may give choroidal vessels free access to the space beneath the RPE and to the subretinal compartment, it may explain the development of a neovascular tissue proliferation in the vicinity of the BM defect. It also raises the question, why not all macular BM defects in highly myopic eyes show an nMMD. It suggests that a BM defect may be a necessary but not sufficient condition for the development of an nMMD. Interestingly, the nMMD prevalence was not associated with the thickness of BM and thickness and density of the RPE and choriocapillaris. It may make one infer that other histomorphometric parameters, biochemical factors or other parameters, may have an influence on the development of an nMMD in eyes with a macular BM defect.

The nMMD prevalence was not significantly correlated with the thickness and density of the choriocapillaris. This finding differs from observations made by McLeod and colleagues and by Sohn and associates in eyes with age-related macular degeneration (AMD)^16–18^. Using Ulex europaeus agglutinin-I labeling, Sohn et al. examined human donor maculae and detected a choriocapillaris loss in eyes with early AMD. The choriocapillaris loss was even more marked in eyes with late AMD with geographic atrophy. McLeod and colleagues made similar observations. A potential limitation of our study as compared to the investigations by Sohn and McLeod is that we did not use a marker for vascular endothelial cells.

Interestingly, the nMMD prevalence was not associated with the thickness of BM and thickness and density of the RPE. It may suggest that other histomorphometric or biochemical factors may be of importance for the development of an nMMD.

In one eye with two nMMDs in close vicinity to each other, the region interposed between these two regions contained an intact BM covered with a de-pigmented single-layered RPE. The RPE cells were in contact with some photoreceptors (Fig. 4). This region may clinically represent a patchy atrophy as part of the clinical definition of category 3 of myopic maculopathy. Patchy atrophies show an areolar loss of pigmented RPE, either without a BM defect, or with a centrally located BM defect^19^. If the assumption is valid that the region between the two nMMDs clinically represented a patchy atrophy, one may infer that this type of patchy atrophy contained RPE cells. These RPE cells, due to their depigmentation, were no longer visible upon ophthalmoscopy. In addition, this region also contained photoreceptors, with markedly shortened, or missing, outer and inner segments (Fig. 4).

When discussing the observations made in the present study, its limitations have to be considered. First, the study does not allow to draw conclusions on the prevalence of nMMD since the study population was selected, the orientation of the histological slides was dependent on the location of the tumors, and only one section per eye was available. An nMMD might thus have been missed even in slides with a horizontal orientation. Second, since the globe sectioning was not focused on the nMMD location, the available section might not have included the center of the nMMD. Third, the investigation consisted of globes with disorders as the cause of their enucleation. These diseases such as secondary angle-closure glaucoma and malignant choroidal melanoma might, directly or indirectly, have influenced the macula, even if eyes with a choroidal tumor at the posterior pole were excluded from the study. Fourth, swelling of the tissue shortly after removal of the globes and shrinkage of the tissue due to the fixation of the tissue and the histological processing of the slides changed the original dimensions of the tissues, so that the measured data do not represent the original dimensions. Fifth, the histomorphometric measurements were performed using a micrometer scale built into the microscope ocular. More sophisticated digital techniques were not applied.

In conclusion, nMMD was characterized by a fibrous pseudo-metaplasia of the RPE and it was associated with macular BM defects, without other detected histomorphometric differences in thickness or density of the RPE, BM and choriocapillaris. The macular BM defect may be a necessary but not sufficient causal factor for the development of the nMMD.
